# Evaluation and assessment of the survival of tooth implant supported prosthesis in tooth and implant supported rehabilitation cases with metal frameworks

**DOI:** 10.1186/s12903-024-04117-9

**Published:** 2024-03-22

**Authors:** Joshua Narde, Nabeel Ahmed, Maria Maddalena Marrapodi, Yuliia Siurkel, Vincenzo Ronsivalle, Marco Cicciù, Giuseppe Minervini

**Affiliations:** 1 Saveetha Dental College and Hospitals, Saveetha Institute of Medical and Technical Sciences (SIMATS), Saveetha University, Chennai, Tamil Nadu, India; 2 Multidisciplinary Department of Medical-Surgical and Odontostomatological Specialties, University of Campania “Luigi Vanvitelli”, Naples, 80121 Italy; 3 Department of Prosthodontics, Saveetha Institute of Medical and Technical Sciences, Saveetha Dental College and Hospital, Saveetha University, Chennai, India; 4 Department of Woman, Child and General and Specialist Surgery, University of Campania “Luigi Vanvitelli,”, Naples, 80138 Italy; 5 International European University School of Medicine, Akademika Hlushkova Ave, 42В, Kyiv, Kyiv, 03187 Ukraine; 6 Department of Biomedical and Surgical and Biomedical Sciences, Catania University, Catania, 95123 Italy

**Keywords:** Dental implant(MESH term), Fixed dental prosthesis, Tooth implant supported prosthesis

## Abstract

**Introduction:**

Over the years, implant therapy has been a commonly used treatment option for individuals who are partially or totally edentulous, with a long-term success rate of over 90%. With significant advancements in biomaterials and technology, implant dentistry can now conduct prosthetic rehabilitations in the majority of patients catering to all types of needs. However, in order to meet the demands of a patient base that is always growing, new trends in implantology are emerging in recent years that are focused on minimally invasive surgery and financial sustainability. In certain clinical scenarios, connecting teeth and implants to support fixed partial prosthesis (FPPs) may be a predictable and workable course of treatment.

**Materials and methods:**

22 patients were selected for this study who had tooth and implant supported prosthesis placed as a final restoration. Out of these 22 patients; 12 were male and 10 were female patients. Implants were placed following proper protocol and if grafting procedures were required they were carried out. A second stage surgical procedure was carried out and delayed loading protocols were followed. The statistical analysis was done using the IBM SPSS 24.0, Chicago, USA. The survival of the implants and teeth were measured by the Kaplan Meier survival scale. Bone loss was assessed at baseline(upon loading), 12 months and 24 months.

**Results:**

The implant survival rate was measured at 6 months, 12 months, 18 months and 24 months. At 24 months, one implant showed failure, so the survival rate of the implants were 95.4%. Bone loss of 1 mm was seen around one implant at 12 months. Bone loss of 1 and 2 mm was present around two implants and one implant respectively at 24 months.

**Conclusion:**

From the results of this study, we can conclude that tooth implant supported prosthesis show very good survival when used in rehabilitation cases.

## Introduction

Over the years, implant therapy has been a commonly used treatment option for individuals who are partially or totally edentulous, with a long-term success rate of over 90% [1–3]. With significant advancements in biomaterials and technology, implant dentistry can now conduct prosthetic rehabilitations in the majority of patients catering to all types of needs(Hong & Oh, 2017). However, in order to meet the demands of a patient base that is always growing, new trends in implantology are emerging in recent years that are focused on minimally invasive surgery and financial sustainability. In certain clinical scenarios, connecting teeth and implants to support fixed partial prosthesis (FPPs) may be a predictable and workable course of treatment [4]. Clinical and statistical analyses of fixed prosthesis supported by tooth and implants have been reported in dental implant-based literature since the mid-1980s by Ericsson and Koth [5]. Providing the patients with these prostheses has led to a significant difference in the quality of life index and patient satisfaction [6]. Patients can now masticate efficiently which at one point for them seemed impossible. The process of osseointegration helps in implants forming a strong bond with the underlying bone. On the other hand, the periodontal ligament supports teeth which allows for physiologic movement that can result in 50–200 μm of displacement of the crown when stresses of 0.1 N are applied [7]. Implants show a maximum displacement of 10 μm [8, 9]. The restoration, abutments, and implant are subjected to an excessive load as a result of the altered behavior of the masticatory forces. An altered force transmission can be noted on the implant, abutment and restoration. In the case of teeth connected to implants, several studies have demonstrated positive outcomes, reporting longitudinal clinical data on treatment outcomes comparable to freestanding implant rehabilitations [10, 11]. However, when tooth-implant supported fixed prostheses were analyzed, other studies showed increased complications and decreased survival rates [12, 13]. There have been reports of complications like tooth intrusion [12], caries at the margin of the crown and fractures of mechanical components [8]. This has led to varying opinions among clinicians and is a highly debatable topic.

The aim of this study is to evaluate the survival of tooth and implant supported prosthesis, the abutments and the implant and to check the viability of these restorations in tooth and implant rehabilitation cases.

## Materials and methods

22 patients were selected for this study who had tooth and implant supported prosthesis placed as a final restoration. Out of these 22 patients; 12 were male and 10 were female patients. The ethics of the Helsinki Declaration were followed and only after the patients gave consent was the treatment started. The format of this study was reviewed by the Institute Review Board at Saveetha Dental College. The inclusion criteria for this type of study was very important in assessing the outcomes. The patients selected to receive this prosthesis had to present with the following clinical findings. The abutment teeth should display good periodontal support which was checked using the Plaque Index and Russel’s Periodontal Index, adequate crown-root ratio ranging from 1:1 to 1:2. A minimum crown height of 6 mm was mandatory for these teeth. The teeth could be either vital or endodontically treated. If endodontic treatment was carried out, a proper apical seal should have been obtained. If posts(metal, fibre or custom-made posts) were placed, the teeth were not selected for the study. Since smoking is considered to be a risk factor in the long term survival of implants, only non smoking patients were included in this study. Those patients who gave a history of parafunctional habits such as bruxism or clenching and other temporomandibular diseases were excluded too. The antagonist arch could contain natural teeth or if treated with restorations, the materials selected were the same for both prostheses.

To select the cases for implant placement, a stringent protocol was followed too. Only cases where two or more teeth were required to be replaced were considered. Adequate amount of bone should have been present to place a 3.5 mm diameter implant which was the narrowest diameter which was allowed for the study. A minimum height of 10 mm was required to place the implant in the posterior maxilla and if sinus augmentation procedures were required, then they were carried out. Only exception was seen in the mandibular arch; if inadequate height was noted beyond the mental foramen, a short implant of minimum length 8.5 mm was placed. Subsequently in these scenarios, wider diameter implants were chosen. Healthy patients were chosen with no absolute contraindication for the implant placement.

### Stage 1 implant surgery

**Fig. 1:**
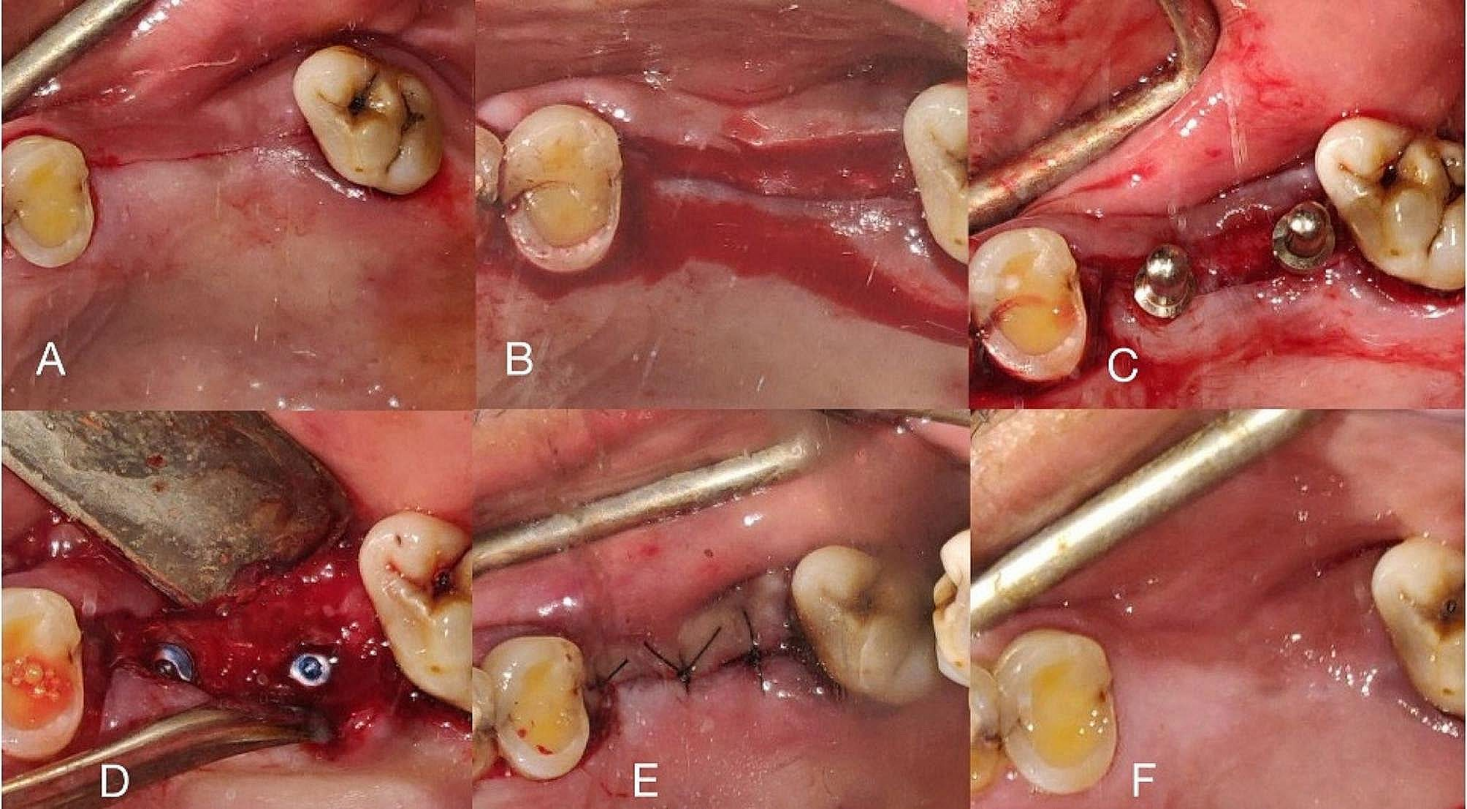
(**A**) Mid-crestal incision, (**B**) Flap elevation, (**C**) Placement of PID markers, Implant placement with cover screw, (**E**) Sutures placed, (**F**) Healing after one month

Prior to the surgical procedure, diagnostic casts were articulated to assess the available occlusal relationship. Cone beam computed tomography(CBCT) scans( Carestream 9600) were carried out to calculate the amount of height and width available for the implant placement. Based on these measurements, correct implant size was selected. The placement procedure was carried out in the following way. A mid crestal incision was given along the edentulous site. Sulcular incisions were done at adjacent teeth to preserve the papilla. A full thickness flap was elevated. Once done, the drilling protocol was carried out as per the instructions provided by the implant manufacturer keeping in mind the drilling speed, torque and the irrigation technique. Once the osteotomy site was completely prepared, the implants were placed and a maximum primary stability of 35Ncm was obtained. A cover screw was then placed. The surgical site was closed using Polyamide 4 − 0 sutures(Healthium Trulon Suture 4 − 0). Simple interrupted sutures or horizontal mattress sutures were placed (Fig. 1A-E). Once hemostasis was achieved, the patient was allowed to leave. The medication prescribed included antibiotics(amoxicillin 500 mg) and ketorloac 10 mg. Suture removal was done after 7 days. This protocol was followed for the implant placements in the maxilla and mandible. In those cases where sinus augmentation procedures were followed, either osteotomes or the hydraulic lift was carried out. The sinus was packed with a combination of autogenous bone which was obtained, blood, xenogenic bone graft material Bio-oss (Geistlich) and ringers lactate solution. The same sequential drilling protocol was followed and the procedure was followed and the implants were placed.

### Stage 2 surgery

**Fig. 2:**
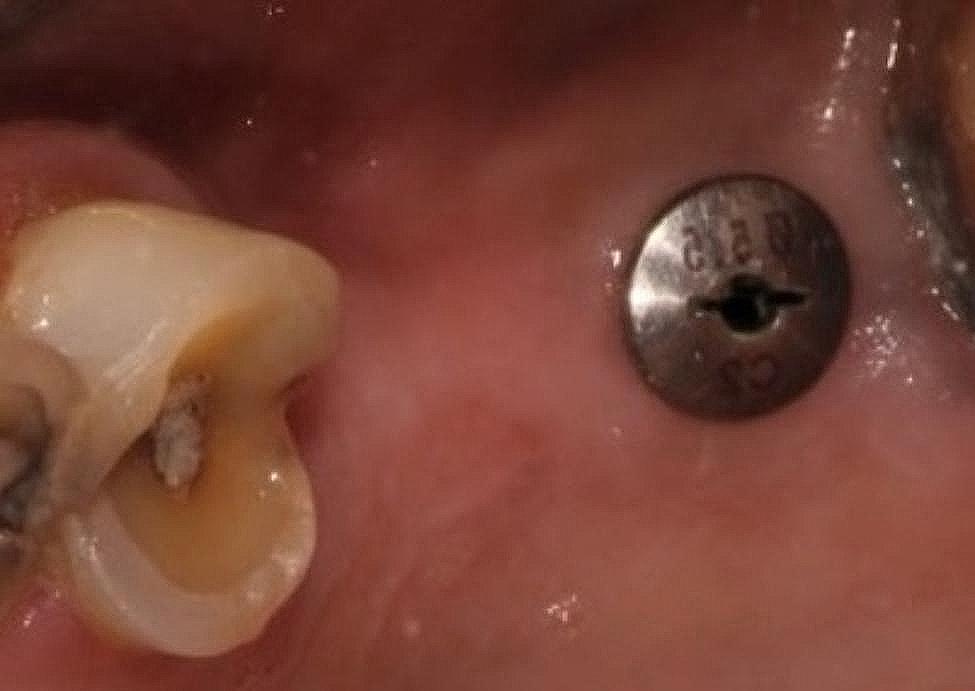
Healing cap placed

In the cases where implants were placed without augmentation procedures, the sites were revisited for the next procedure after 3 months whereas those that needed augmentation were opened only after 5–6 months. Healing caps were then placed for a minimum of 15 days. Adequate sized healing abutments were selected to achieve a good emergence profile. If necessary, customization was carried out(Fig. 2).

### Prosthetic phase

**Fig. 3:**
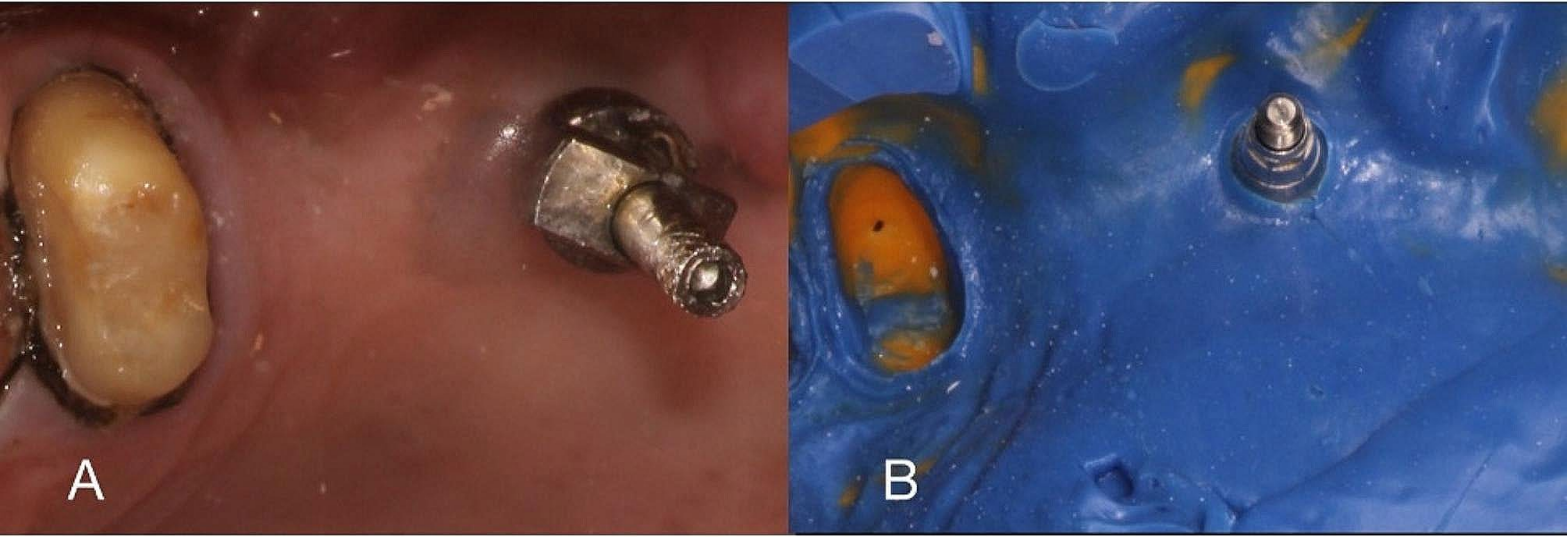
(**A**) Tooth preparation followed by cord packing, (**B**) Final impression

**Fig. 4:**
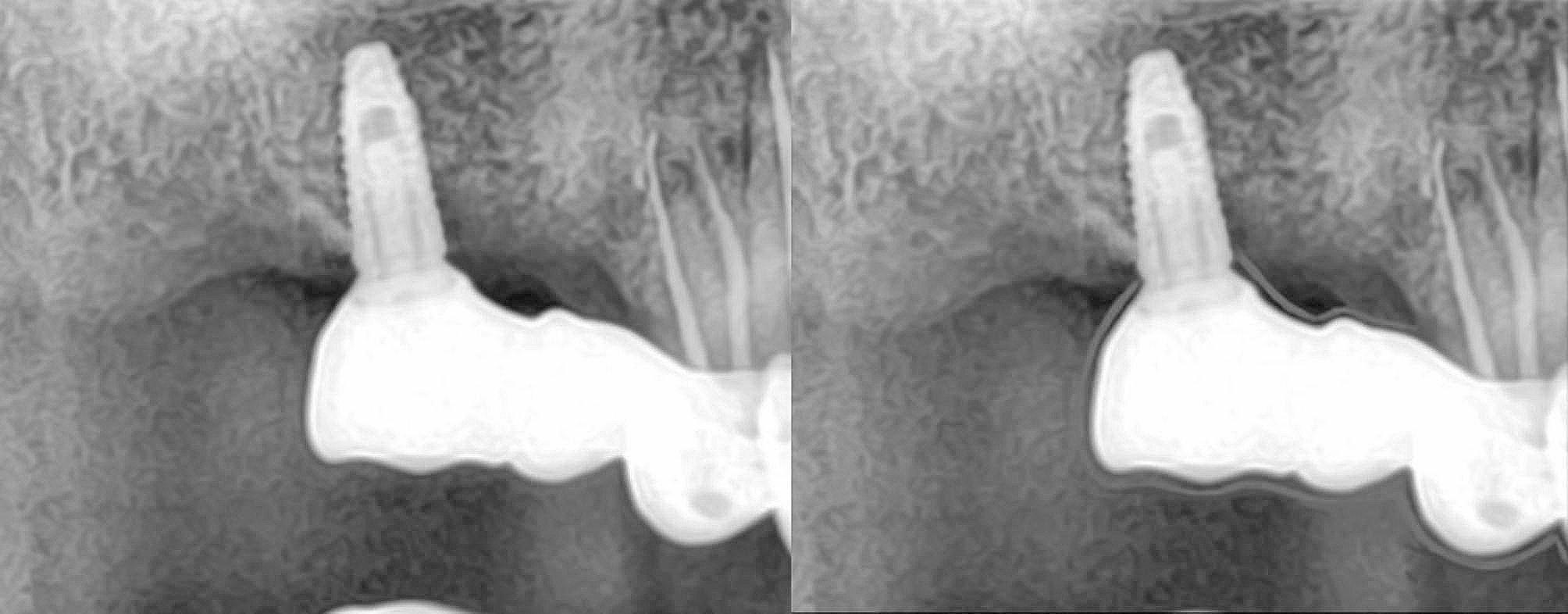
(**A**) Coping trial, (**B**) Post cementation

After sufficient healing time for the gingiva was given, the prosthetic phase was started. Closed tray impression copings were chosen for the implant based on the size.These impression posts were radiographically verified before taking the impression. The adjacent teeth were prepared using diamond points (Mani, INC). Occlusal reduction of 1.5-2 mm was done, margins were either chamfer or shoulder and a convergence of 6–10 degrees was achieved. Most importantly proper smoothening and roundening of the line angles was done. Once the preparation was complete, 2 retraction cords ( SURE-Cord) of sizes 000 and 0 were packed using a cord packer(Hu Friedy). The cords were dipped in a hemostat (Prevest Hemostal) before the placement. Only once a dry field and hemostatsis was achieved was the impression made. The upper cord was removed first. An addition silicone (Zhermack elite HD+) impression was then made using putty and light body(Fig. 3A-B). Once fully set, the impression was then removed. It is assessed by checking a 360 degree flash around the prepared abutment tooth. The impression coping is then unscrewed and placed into the impression. The click sound signifies reseating of the post in the correct orientation. The impression is then disinfected using 70% isopropyl alcohol. A cast is poured using Type IV die stone (Zhermack) and allowed to set. Once set, the cast was retrieved and examined for air bubbles or voids. The next step was the designing of the framework for the prosthesis. software(Exocad 2.4 Plovdiv) was used and the copings were designed. Once designed, they were then milled in wax(Roland DGShape, DWX 52D) and later cast using cobalt chrome. The copings were evaluated and radiographically verified (Fig. 4A). Upon radiographic confirmation of proper marginal fit, finishing was carried out. The veneering of ceramic(SHOFU Vintage Pro) was done post the finishing. Occlusal corrections were made during the bisque stage after intraoral checks and the final glaze was applied. A final torque of 20 Ncm was given before the cementation. The restorations were then luted using glass ionomer cement( SHOFU HY-BOND Glass Ionomer). Excess cement was cleaned and the final occlusion was checked(Fig. 4B).

### Follow up examinations

**Fig. 5:**
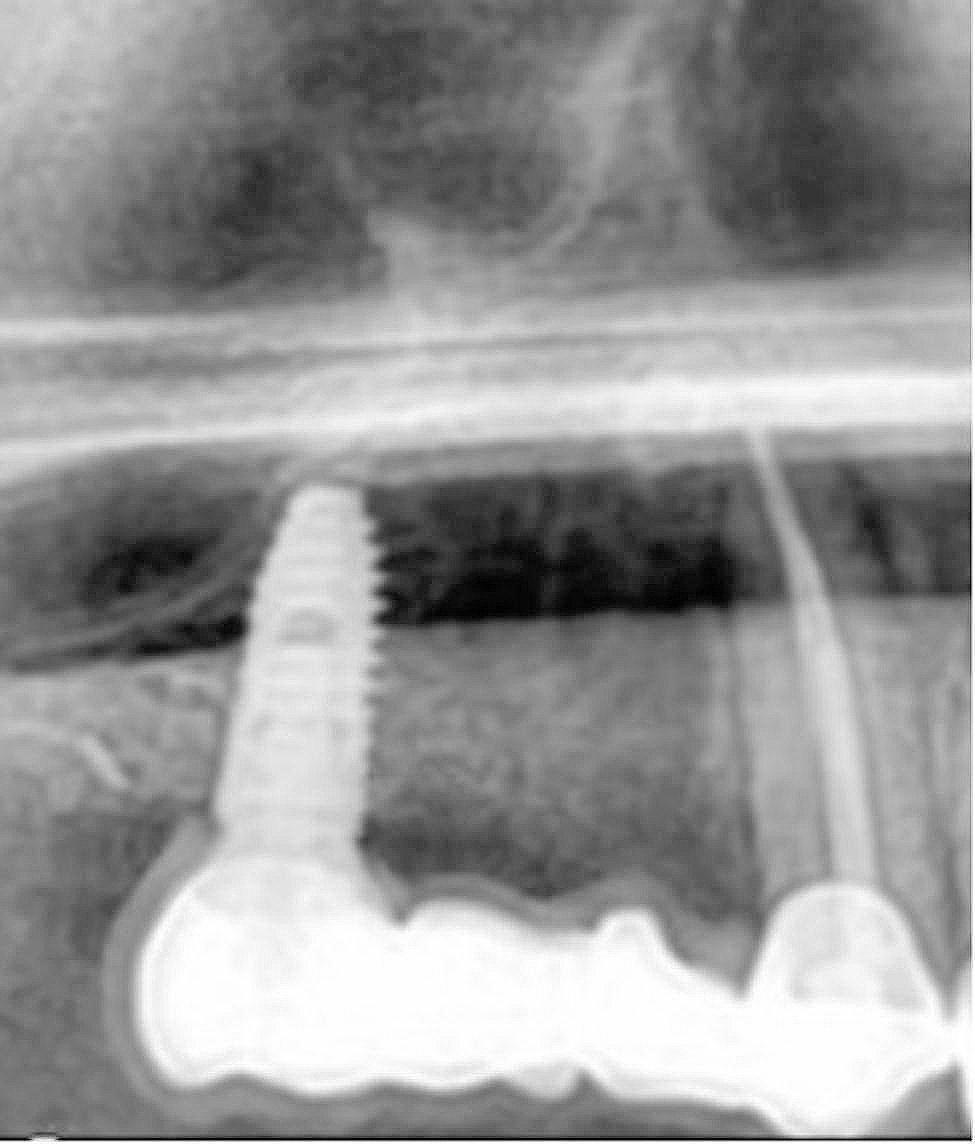
(**A**) Radiograph at baseline, (**B**) Radiograph at 12 months showing 1mm bone loss, (**C**) Radiograph at 24 months showing 1mm bone loss

The baseline to evaluate the prosthesis was checked 7 days after the cementation. At this time, intraoral photographs were taken, probing depth was measured and a radiograph was taken to ascertain the bone level at the time of loading. The patient was recalled at timely intervals of 6 months, 12 months, 18 months and 24 months. An intraoral picture and a radiograph was taken at every appointment to compare with that at the baseline. A standardization was done for the two-dimensional radiographs which would help us in proper evaluation [14]. This made the process of evaluating the radiographic changes if any, more easier (Fig. 5).

### Statistical analysis

The statistical analysis was done using the IBM SPSS 23.0, Chicago, USA. The survival of the implants and teeth were measured by the Kaplan Meier survival scale. Bone loss was assessed at baseline(upon loading), 12 months and 24 months using repeated measures ANOVA Test.

## Results

For this study, descriptive and analytical statistics were done. The data is represented in number and percentages and mean with standard deviation. The Kaplan-Meier survival analysis was performed. The level of significance was kept at *p* < 0.05.

- The implant survival rate was measured at 6 months, 12 months, 18 months and 24 months. At 24 months, one implant showed failure, so the survival rate of the implants were 95.4%(Table 1).
- The survival rate of tooth was 100% at 6 and 12 months. At 18 months, one tooth showed failure, so the survival rate of tooth group at 18 months was 96%. At 24 months, one more tooth showed failure, so the survival rate at 24 months was 92% (Table 2).
- The survival rate of implant prosthesis was 100% at 6 and 12 months. At 18 months, one implant prosthesis showed failure, so the survival rate of implant prosthesis at 18 months was 95.4%. On evaluation at 24 months, one more prosthesis showed signs of failure, so the rate was seen at 90.9% (Table 3).
- **Kaplan-Meier survival analysis test results** The mean survival time of implant group was 24.00 ± 0.00 months and of tooth group was 23.52 ± 1.66 months. The Log Rank (Mantel-Cox) test was not statistically significant (*p* = 0.355). Graph 1 depicts the Kaplan-Meier survival curve.
- **bone loss around implants** Bone loss of 1 mm was seen around one implant at 12 months. Bone loss of 1 and 2 mm was present around two implants and one implant respectively at 24 months(Table 4).

**Table 1:** The implant survival rate was measured at 6 months, 12 months, 18 months and 24 months. At 24 months, one implant showed failure, so the survival rate of the implants were 95.4%(

Timeline	Implant Survival		Survival Rate
6 months	22 (100.0)	0 (0.0)	100%.
12 months	22 (100.0)	0 (0.0)	100%
18 months	22 (100.0)	0 (0.0)	100%
24 months	21 (95.4)	1 (4.5)	95.4%

**Table 2:** The survival rate of tooth was 100% at 6 and 12 months. At 18 months, one tooth showed failure, so the survival rate of tooth group at 18 months was 96%. At 24 months, one more tooth showed failure, so the survival rate at 24 months was 92%

Timeline	Tooth Survival		Survival Rate
6 months	25 (100.0)	0 (0.0)	100%
12 months	25 (100.0)	0 (0.0)	100%
18 months	24 (92.0)	1 (4.0)	96%
24 months	23 (88.0)	2 (8.0)	92%

**Table 3:** The survival rate of implant prosthesis was 100% at 6 and 12 months. At 18 months, one implant prosthesis showed failure, so the survival rate of implant prosthesis at 18 months was 95.4%. On evaluation at 24 months, one more prosthesis showed signs of failure, so the rate was seen at 90.9%

Timeline	Prosthesis Survival		Survival Rate
6 months	22 (100.0)	0 (0.0)	100%
12 months	22 (100.0)	0 (0.0)	100%
18 months	21 (95.4)	1 (4.5)	95.4%
24 months	20 (90.9)	2 (9.1)	90.9%

**Table 4:** Bone loss of 1 mm was seen around one implant at 12 months. Bone loss of 1 and 2 mm was present around two implants and one implant respectively at 24 months(Table 4)

Timeline	Bone loss in mm		
	Baseline	12 months	24 months
Baseline	22 (100.0)	0 (0.0)	0 (0.0)
12 months	21 (95.4)	1 (4.5)	0 (0.0)
24 months	19 (86.4)	2 (9.1)	(4.5)

## Discussion

Tooth implant supported prosthesis is considered to be a highly controversial and debatable topic when different types of implant restorations are discussed. The main objective of this study was to assess the survival of these prosthesis when used in rehabilitation cases. From the results of the current study, tooth implant supported prosthesis could be seen as a promising restoration.

From the current available literature, implant supported fixed dental prosthesis have a survival rate of 98.3% when metal ceramic is used as the material of choice and 93.0% when zirconia and ceramic is used for a period of 5 years [15]. Ceramic related complications are consdiered to be more in cases of zirconia based prosthsies(4.1%) and lesser in metal ceramic prosthesis(0.3%).Since the advent of zirconia-based reconstructions, chipping of the zirconia veneering ceramic has been a commonly reported issue. According to a systematic review, 54% of zirconia veneering ceramic chipping occurs at tooth-supported reconstructions [15, 16]. Research on zirconia FDPs supported by implants has shown rates as high as 50% [17]. Although the high incidence of chipping that was initially experienced has decreased due to advancements in zirconia veneering ceramics and veneering procedures, the issue still remains as the main technical complication [18]. Zirconia based studies might show enhanced esthetics but also come with issues such as delamination and chipping [19]. Hence metal ceramic still remains to be the gold standard when implant prosthesis are chosen. The results depicting the high survival rate from this study also prove how effective metal ceramic prosthesis can be when considered to be a material of choice when connecting tooth and implants. Monolithic zirconia can be considered to be a material to be used for implant supported restorations since they have shown lesser failure [20] but more research is required to be done in relation to tooth and implant supported prosthesis.

One of the complications seen with teeth and implants connected splinted to each other is intrusion. Tooth intrusion is a complex condition that can result from a variety of factors, including mechanical binding, mandibular flexion and torsion, flexion of the fixed partial denture, impaction of debris and parafunctional activity, impaired rebound memory, and significant energy dissipation caused by the elastic and inelastic deformation of the periodontal ligament [19]. This was claimed to be one of the reasons to avoid using these restorations. The use of non-rigid connector was supposed to be the solution to such a problem [21]. Evidence related to the use of such connectors has been less and ambiguous too. Our study incorporated the use of a rigid framework. Srinivasan et al. in his study has also stated that the connectors of choice should be rigid and that using non rigid connectors might cause more deleterious effects to the prosthesis [22]. This has been backed up by other studies too [23–25]. The results obtained from this study are in accordance with the claims made and that neither was the survival affected nor intrusion seen in cases. The type of prosthesis chosen was cement retained prosthesis and studies show that there was significant difference between screw-retained and cement-retained prosthesis [26]. The decision to use permanent cement retained prosthesis has been backed by Boekcler et al. who states that the cement used can prevent intrusion [27].

Prosthesis related technical complications were seen in the form of ceramic chipping and fracture which has also been noted in literature. This could be due to the varying amounts of occlusal forces acting on the restoration. The type of forces and direction of action of forces could be the reason for the ceramic chipping [28]. Esthetic changes in the prosthesis were not seen even though metal ceramic was used as the material of restoration and not zirconia.

The marginal bone loss was measured by checking the bone levels radiographically and comparing it with the condition at the time of loading. This was recorded at 12 months and 24 months. The maximum amount of bone loss seen was 2 mm at 24 months in one case where the prosthesis also showed technical complications. This could be due to improper occlusal scheme at the time of loading or heavier occlusal loads being transferred [28, 29]. In other studies that were conducted checking the marginal bone loss levels between implant supported FPDs and tooth implant supported FPDs, it was noted that the margianl bone loss was more in the implant supported group [30] and hence using the tooth and implant connection could be seen as a viable prosthetic option with good results. Secondary caries was seen in three teeth at 18 months and 24 months. One tooth was endodontically whereas two were not. The vital teeth showed caries and endodontic intervention was required for the same. No significant difference was seen between those abutments that were vital and root canal treated. Periodontal complications were not seen in any of the cases which included deep pockets or increased bleeding on probing. Patients were explained the importance of oral hygiene to be followed at home and also the importance of follow-up appointments.

From the evidence that is available to us, it can be seen that our results fall well into the range that is given by other authors in their clinical trials. In a study conducted by Beuer et al. [31], the success of zirconia-based fixed dental prosthesis connecting an implant to a tooth was assessed. The results obtained displayed a similar performance in terms of the survival rates of both the teeth and the implants up to 93.5% up to 40 months with a rate of 14.5% chances of chipping taking place during this period. The mean survival rates of tooth supported FPDs in the same duration is between 90 and 100% and implant supported FPDs are upto 95%. This goes to show that the survival rates are similar. To further strengthen our claims, Rammelsberg et al. in his study also reached similar conclusion [32–41]. The use of metal ceramic framework led to high survival rates in the cases where veneering material was used. The discussion to use monolithic zirconia frameworks was also brought up although lesser studies and evidence was available and those which are present demonstrated complications with the framework. The use of metal-ceramic is still considered safer and shows promising results too.

In a systematic review by Taneja et al. in 2023 [42], it was stated that no significant difference was seen in the survival comparison between tooth and implant supported FPDs and implant-supported FPDs.

A drawback of this case series is that temporization was not done for the implants and only healing caps were placed. A longer follow-up would be required for more studies following these patterns of restorations.

## Conclusion

Tooth implant supported prosthesis is done mainly when the clinician has no other option left in providing a fixed restoration. These types of prosthesis provide a good short and long-term solution for the same with less incidences of failure. The metal ceramic restorations with rigid connectors can be seen as the material and design of choice for the prosthesis. From the results of this study, we can conclude that tooth implant supported prosthesis show very good survival when used in rehabilitation cases.

## Data Availability

The data will be available on reasonable request from the corresponding author.
